# Drug Induced Thrombotic Microangiopathy with Certolizumab Pegol

**DOI:** 10.4274/balkanmedj.2017.1224

**Published:** 2018-09-21

**Authors:** Mehmet Baysal, Elif Gülsüm Ümit, Fatih Sarıtaş, Nil Su Kodal, Ahmet Muzaffer Demir

**Affiliations:** 1 Department of Hematology, Trakya University School of Medicine, Edirne, Turkey; 2 Clinic of Rheumatology, Tekirdağ State Hospital, Tekirdağ, Turkey

**Keywords:** Certolizumab pegol, drug-induced, etiology, thrombotic microangiopathies

## Abstract

**Background:**

Certolizumab pegol is used to treat ankylosing spondylitis, Crohn’s disease, psoriatic arthritis, and rheumatoid arthritis. Unlike other monoclonal antibodies such as infliximab and adalimumab, certolizumab does not contain an Fc fraction and hence does not induce complement activation. In this report, we describe the case of a patient with thrombotic microangiopathy caused due to certolizumab pegol, with a brief description about the pathophysiological approach to thrombotic microangiopathy.

**Case Report:**

A-39-year-old man suffering from ankylosing spondylitis for the past 10 years presented with fatigue. He had been on certolizumab pegol treatment for 6 months, starting with 400 and 200 mg every 2 weeks. He had significant nonimmune hemolytic anemia and thrombocytopenia without a disseminated intravascular coagulopathy. Schistocytes were observed in more than 10% of the erythrocytes per field. Plasma exchange along with corticosteroid treatment was started. There was a dramatic improvement within a week, and after 10 sessions of plasma exchange, the patient was discharged on corticosteroids with a tapering plan. ADAMTS13 enzyme activity was determined to be normal.

**Conclusion:**

The development of drug-induced thrombotic microangiopathy may be either immune-mediated or dose-dependent toxicity-mediated Anti-drug antibodies and their immunological aspects are still unclear and yet to be elucidated.

Certolizumab pegol is used to treat ankylosing spondylitis, Crohn’s disease, psoriatic arthritis, and rheumatoid arthritis. It is a compound consisting of the Fab fragment of the tumor necrosis factor alpha monoclonal antibody and polyethylene glycol. The addition of polyethylene glycol reduces the antigenicity of the compound extends the half-life. Unlike other monoclonal antibodies such as infliximab and adalimumab, certolizumab does not contain an Fc fraction and hence does not induce complement activation, antibody-dependent cell-mediated cytotoxicity, or apoptosis. The best known adverse effects of certolizumab are nausea, antibody development against the molecule, and infections. Drug-induced thrombotic microangiopathy (DITMA) with monoclonal antibodies is rare. Cetuximab and bevacizumab, both growth factor inhibitors used in the treatment of solid tumors, were reported to be associated with DITMA with a probable explanation of toxicity-mediated mechanism.

In this report, we describe the case of a patient with thrombotic microangiopathy caused due to certolizumab pegol, with a brief discussion on the pathophysiological approach to thrombotic microangiopathy.

## CASE PRESENTATION

A 39-year-old man suffering from ankylosing spondylitis for the past 10 years presented with fatigue. He denied having fever, nausea, vomiting, diarrhea, confusion, or headache. He had been on certolizumab pegol treatment for 6 months, starting with 400 and 200 mg every 2 weeks. His initial complete blood count showed the following results: hemoglobin 14.4 g/dL, hematocrit 52%, leucocyte count 8900/mm^3^, and platelet count 415000/mm^3^. His monthly complete blood count evaluation was totally normal till 5 months after the start of the treatment, and 1 month before our evaluation, the hemoglobin level was 11.4 g/dL; however, the patient was asymptomatic and this result was ignored. He denied any recent travel or consuming illicit drugs or quinine-containing drugs or water.

His current complete blood count showed the following results: hemoglobin 5.3 g/dL, Htc 16.1%, mean corpuscular volume 90 fL, leucocyte count 4460/mm^3^, and platelet count 28000/mm^3^. Coagulation profile was normal with a negative D-dimer result. Biochemical analysis showed a significant elevation of lactate dehydrogenase level of 5680 U/L (upper limit of normal: 220 U/L), with a total bilirubin of 2.2 mg/dL, direct bilirubin of 0.32 mg/dL, and haptoglobulin of 28 mg/dL (lower limit of normal: 30 mg/dL). Renal functions were normal, and there was no proteinuria. Peripheral blood smear showed schistocytes in almost 20% of every erythrocyte per field. The patient was considered as having thrombotic microangiopathy, and plasma exchange along with 1 mg/kg/day of methylprednisolone were started. Before plasmapheresis, samples were collected for ADAMTS13 evaluation. With a daily plasma exchange and corticosteroid treatment and cessation of certolizumab, there was a rapid and dramatic improvement in the clinical and laboratory results of the patient, and after 10 consecutive exchange sessions, the plasma exchange was stopped, and the patient was discharged on corticosteroid treatment alone. No relapses were observed, and the corticosteroid treatment was rapidly tapered and stopped. ADAMTS13 activity was found to be 86%, which was normal as expected. Written informed consent was obtained from our patient for publishing this case report. The follow-up results of our patient are summarized in Table 1.

## DISCUSSION

Thrombotic microangiopathy may be categorized as primary or secondary. Primary thrombotic microangiopathy syndromes include thrombotic thrombocytopenic purpura, ADAMTS13 enzyme deficiency, Shiga toxin-mediated hemolytic uremic syndrome, complement-mediated thrombotic microangiopathy or complement-mediated hemolytic uremic syndrome, DITMA, metabolism-mediated thrombotic microangiopathy as in vitamin B12 metabolism disorders, and coagulation-mediated thrombotic microangiopathy, which are hereditary deficiencies of coagulation proteins. The term atypical hemolytic uremic syndrome is no longer used since it has no specificity. Secondary thrombotic microangiopathy includes pregnancy complications, severe hypertension, systemic infections, malignancies, rheumatic disorders, transplantation, disseminated intravascular coagulation, and severe vitamin B12 deficiency (1,2). As this classification may further be changed, DITMA is classified within primary thrombotic microangiopathy.

The development of DITMA may be either immune-mediated or dose-dependent toxicity-mediated. Examples of the drugs causing the antibodies to react with platelets and endothelial cells are quinine, gemcitabine, oxaliplatin, and quetiapine. Toxicity-mediated dose-dependent thrombotic microangiopathy may develop due to chemotherapeutic agents such as gemcitabine (acting as an antigen itself); immunosuppressive agents such as cyclosporine and tacrolimus; and growth factor inhibitors such as sirolimus, bevacizumab, and cetuximab (1,2,3).

Anti-drug antibodies have been demonstrated with certain monoclonal antibodies such as adalimumab, infliximab, golimumab, etanercept, and certolizumab pegol. These antibodies are generally non-neutralizing and not associated with decreased trough levels of drugs or impaired clinical response. Their clinical impact in terms of their immunogenicity remains unclear. Combined therapy with traditional nonbiologic drugs is associated with decreased levels of such circulating anti-drug antibodies. These antibodies are classified as either human antichimeric antibodies or human antihuman antibodies depending on the origin of the target molecule (4). Treatment with tumor necrosis factor  antibodies is also associated with additional autoimmune processes, other than the ones being treated with these tumor necrosis factor  antibodies. These systemic autoimmune processes associated with a tumor necrosis factor  inhibitor include drug-induced lupus syndromes, vasculitis, sarcoidosis, antiphospholipid syndrome, dermatomyositis, and lung, eye, and hepatic involvement; demyelinating diseases have also been reported to be associated with tumor necrosis factor  inhibitors (5). Though the immune-mediated DITMA is generally expected within 21 days of exposure to the suspected drug, we observed it almost 5-6 months after the start of the treatment. This delay may be due to concomitant use of corticosteroids in the initial symptom relief period.

In our case, based on the normal ADAMTS13 activity results, we emphasize that the mechanism of DITMA development is a probable immune-mediated and autoimmune process caused due to an antibody, although we could not demonstrate the specific anti-drug antibody. The irony with this specific drug is that tumor necrosis factor inhibitors are supposed to have anti-inflammatory effects and, particularly, certolizumab does not activate the complement system, since eculizumab is also used in DITMA treatment on a case-based approach. In our case, an anti-drug antibody may be responsible for the development of thrombotic microangiopathy. Anti-drug antibodies and their immunological aspects are still unclear and yet to be elucidated.

## Figures and Tables

**Table 1:**
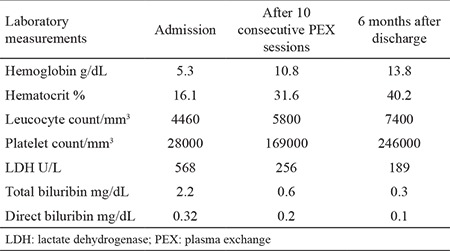
Follow-up and laboratory results of our patient
